# Mammographic Breast Density in Chinese Women: Spatial Distribution and Autocorrelation Patterns

**DOI:** 10.1371/journal.pone.0136881

**Published:** 2015-09-02

**Authors:** Christopher W. K. Lai, Helen K. W. Law

**Affiliations:** Department of Health Technology and Informatics, The Hong Kong Polytechnic University, Hung Hom, HKSAR, China; University of South Alabama Mitchell Cancer Institute, UNITED STATES

## Abstract

Mammographic breast density (MBD) is a strong risk factor for breast cancer. The spatial distribution of MBD in the breast is variable and dependent on physiological, genetic, environmental and pathological factors. This pilot study aims to define the spatial distribution and autocorrelation patterns of MBD in Chinese women aged 40–60. By analyzing their digital mammographic images using a public domain Java image processing program for segmentation and quantification of MBD, we found their left and right breasts were symmetric to each other in regard to their breast size (Total Breast Area), the amount of BMD (overall PD) and Moran's I values. Their MBD was also spatially autocorrelated together in the anterior part of the breast in those with a smaller breast size, while those with a larger breast size tend to have their MBD clustered near the posterior part of the breast. Finally, we observed that the autocorrelation pattern of MBD was dispersed after a 3-year observation period.

## Introduction

According to the American College of Radiology Breast Imaging Reporting and Data System (BI-RADS), contents of breast tissues can be classified as entirely fat, scattered fibro-glandular densities, heterogeneously dense and extremely dense. The distribution of these tissues is variable, and dependent on physiological, genetic, environmental and pathological factors. Women with mammographic breast density (MBD) content greater than 75% of the total area of the breast would have a breast cancer risk five times higher than those with nearly no MBD[1], and therefore it becomes one of the strongest risk factors in the prediction of breast cancer risk[2–7]. Nevertheless, even though MBD has been studied extensively over decades, information on the spatial distribution of MBD in breast is scarce. Also, the presence of MBD often mimic accurate diagnose of small lesions and micro-calcification in mammograms, resulting in a high false-negative and false-positive diagnosis.

To date, screening mammography provides an effective early risk assessment of breast cancer risk in women aged over forty. In 2009, Pereira et al. reported the first study on the distribution of MBD in 165 Caucasian women aged 39–41 [8]. The result of their study demonstrated that MBD within 48 sub-regions of the whole breast was generally clustered together, and the autocorrelation pattern had not changed after an eight-year observation period [7]. The author further indicated in his later study in 2011that most tumours of breast were predominantly detected from the tissues of MBD [6], and the result is consistent with another study showing a strong correlation between MBD and carcinogenesis [9].

Young women usually have a smaller proportion of fat content relative to fibro-glandular tissue in their breasts than older women, and that Chinese women usually have denser and smaller-sized breasts when compared to Caucasian women [10]. It is therefore straightforward to postulate that the variations in breast size and the distribution of MBD between Western and Chinese women may affect the cancer risk diagnosis differently. Related information on the spatial distribution and autocorrelation patterns of MBD has been defined in Western women, but it is still unknown in Chinese women. In this regard, the aim of this study is to define the spatial distribution and autocorrelation patterns of MBD in middle-aged Chinese women using a public domain Java image-processing program for automated segmentation and quantification of MBD.

## Materials and Methods

**Fig 1:**
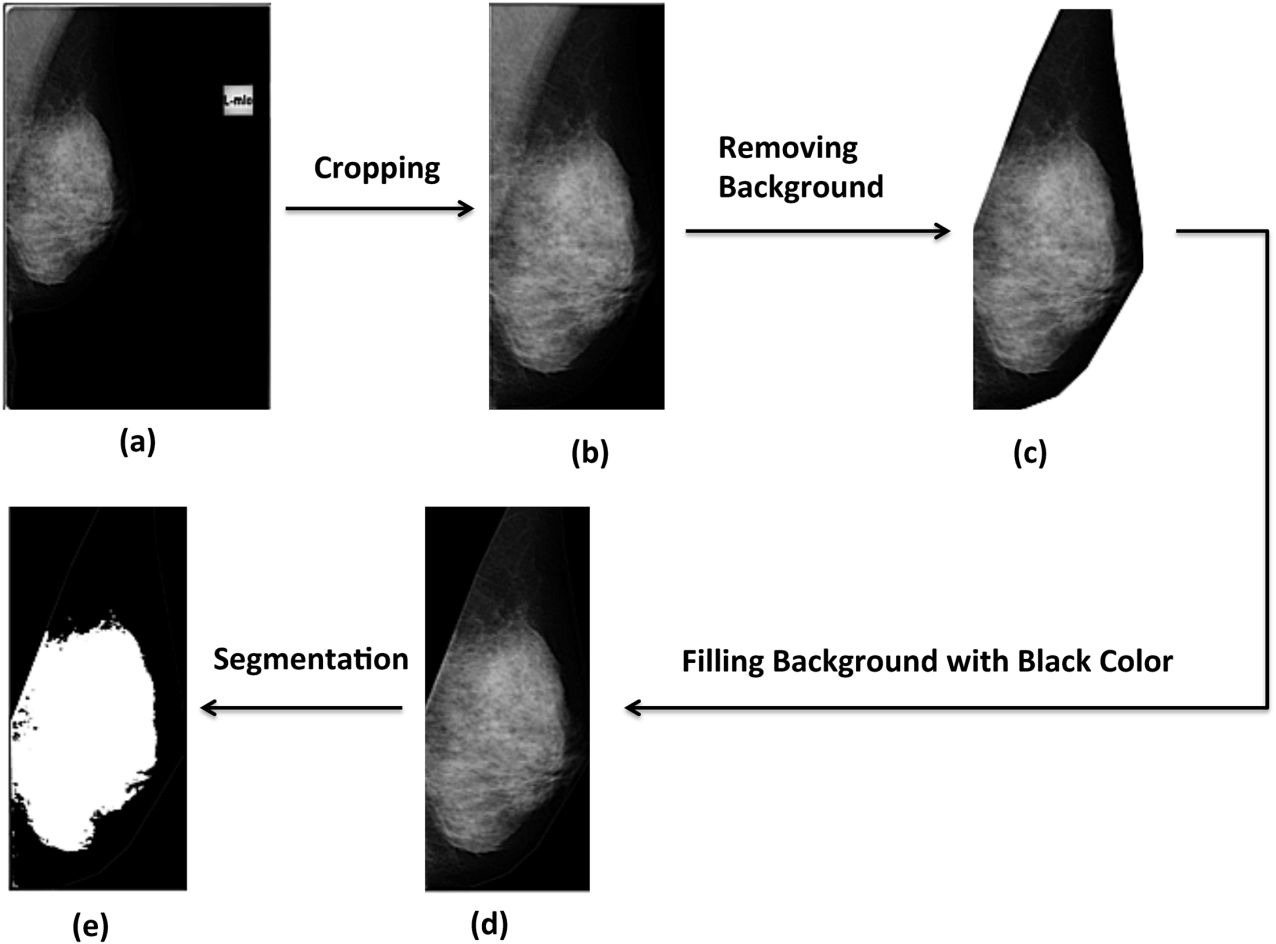
The procedural steps for the segmentation of Mammographic Breast density (MBD) in this study. (a) Original image; (b) Cropping the image to the minimal size; (c) Contouring the pectoral muscle and the breast outline; (d) filling the background outside the area of interest with black color and (e) MBD is segmented and represented as white area in the final processed image.

Digital mammograms (all in DICOM format) in mediolateral oblique (MLO) projection, from 50 Chinese women (yearly body check-up cases) aged from 40–60, were randomly collected retrospectively from the Radiography Clinic of the Hong Kong Polytechnic University (Fig 1a). Three images (one mammogram of the left breast was collected at entry, and bilateral mammograms were collected at exit) were collected from each subject at two time points 3 years apart between entry (2011) and exit mammogram (2014). All patient records and images were collected by one of our research assistants (AF), and the collected information were anonymized and de-identified prior to further analysis.

This study was approved by the Human Subject Ethics Subcommittee of the Hong Kong Polytechnic University. This was a retrospective study and no written/oral consent could be obtained. However, being a clinical teaching and research clinic, all subjects were well informed and agreed that their de-identified personal information and images might be used and reported for teaching and research purposes before they could receive our clinical services at Radiography Clinic.

A total of 150 collected images were processed using the NIH ImageJ program (Version 1.47, Rasband, US National Institute of Health, Bethesda, Maryland, USA). At first, the border of the breast and the margin of the pectoralis muscle were manually defined by another two research assistants (JL and AL) (Fig 1b), who were blinded to the patient and image information. Then, the area outside this defined region of interest was cropped (Fig 1c) and filled with black color (Fig 1d) in order to prevent any radio-dense objects outside the breast region being considered as part of MBD.

Next, the cropped and pre-processed images were segmented into dense and non-dense areas (Fig 1e) with the application of an automated global-thresholding method called Moments (a method that can preserve the moments of the original image in the thresholded result) [11]. This automated segmentation method has shown a good agreement with the gold standard segmentation method called Cumulus method [12,13]. After calibrating the pixel size of the image, the total area of the breast, and the overall percentage area of MBD (overall PD) of the whole breast were defined automatically. Finally, in order to investigate the spatial distribution and autocorrelation patterns of MBD, all segmented images were divided into 48 equally sized rectangular sub-regions (in six columns and eight rows with coordinates) using Split and Tile Image Splitter software (Version 2.11, SoftDD Software) and MATLAB (The MathWorks, Inc.). These fragmented images were generated and re-entered into the NIH ImageJ program for the automatic quantification of percentage area of MBD in each sub-region area by our research assistant (KN), and the result will be described as regional percentage area of MBD (regional PD) with coordinates.

### Data analysis

**Fig 2:**
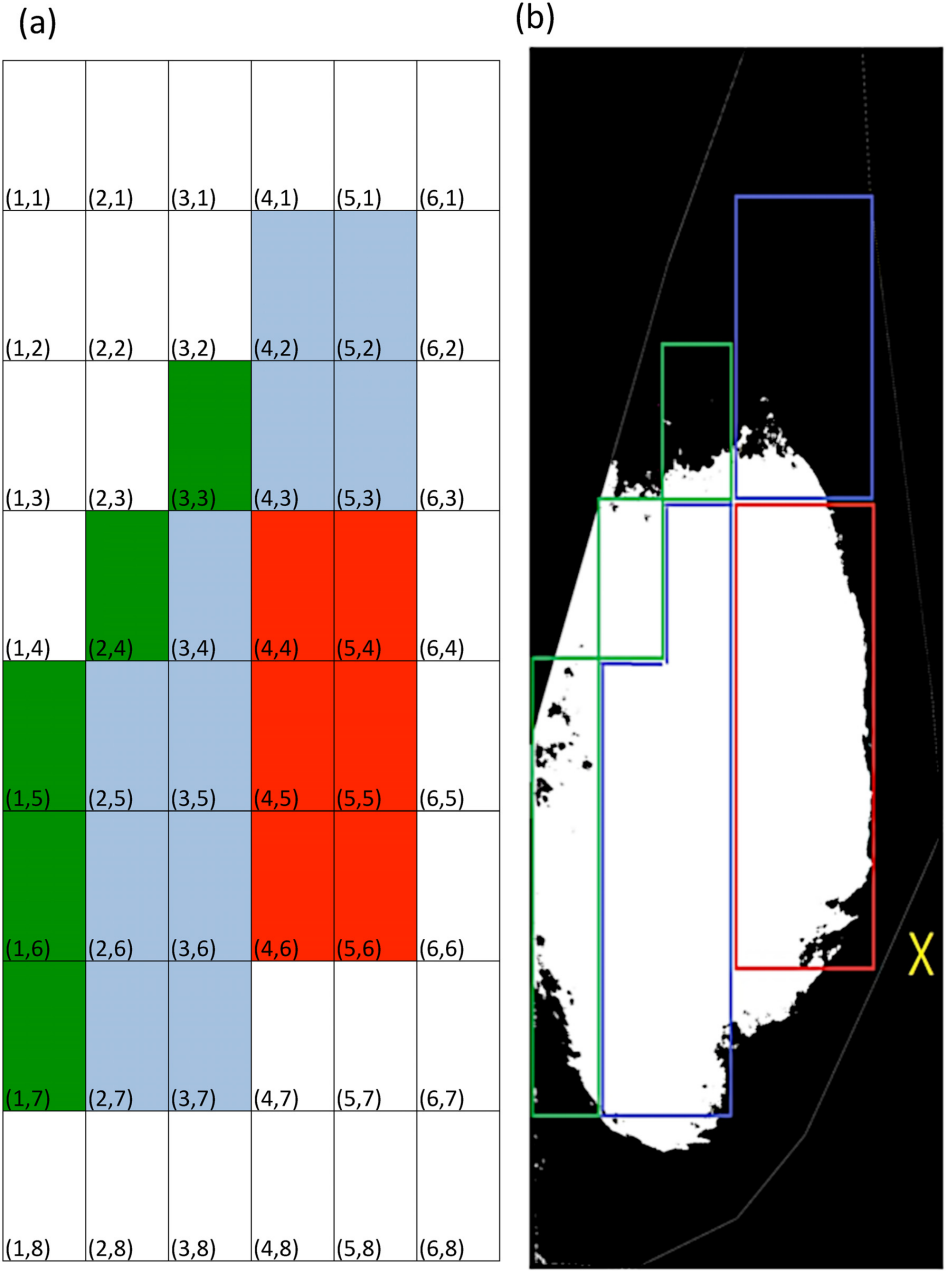
An illustration of the 48 coordinates and the three self-defined zones of the left exit mammogram. (a) The Coordinate system used in the present study divides the whole image into a total of 48 sub-regions. The 3 self-defined zones: anterior, middle and posterior zones of the breast were labeled in red, blue and green respectively. (b) A sample image showing the relative position of different zones. Please note the position of the subject’s nipple was labeled with a "X" mark.

In this study, we divided the 50 subjects into quantiles according to the degree of overall PD value of the Left Exit Mammogram. All presented data were reported as mean ± standard derivation, and were analyzed using IBM SPSS Statistics software (Version 17.0, SPSS Inc., Chicago, Illinois, USA). A *p*-value of <0.05 was considered as significant. To describe the spatial distribution pattern of MBD (Fig 2a and 2b) in our Chinese subjects, the regional PD in the 48 coordinates (Column, Row) and the average of regional PDs under three self-defined regional zones (anterior, middle and posterior zone of the breast) (zonal PD) of the left exit mammograms were presented. Finally, we used Moran's I equation to estimate the autocorrelation pattern over the 48 regional PD values,

$$Moran^{\prime }s\;I=\;\frac{N}{\sum_{i}\sum_{j}w_{ij}}\frac{\sum_{i}\sum_{j}w_{ij}\left(x_{i}-\bar{x}\right)\left(x_{j}-\bar{x}\right)}{\sum_{i}{\left(x_{i}-\bar{x}\right)}^{2}},$$

where the weighting factor *w*_*ij*_ was defined as 1/*d* ^2^, and *d* referred to the distance between the midpoints of adjacent two sub-regions.

## Results

A total of 150 mammograms were collected and analyzed using our proposed semi-automated method. Their age at the time of entry mammogram was 50.3y±4.7. Table 1 summarized their breast size (Breast Area) with the proportion of dense area and lucent area, and the overall percentage area of MBD (Overall PD) in four different groups according to their overall PD at the Left exit mammogram. Our results indicated that overall PD was similar between the left and right breasts (*p* = 0.43), and between the entry and exit mammogram of the left breast (*p* = 0.51).

**Table 1:** Breast size and Overall Percentage of mammographic breast density in entry and exit mammograms.

Grouping according to the Overall PD at exit mammogram (Left breast) by quantiles	Group 1 (<20%)	Group 2 (20–40%)	Group 3 (40–60%)	Group 4 (60–80%)	All groups
**No of Subjects**	12	16	15	7	50
**Age at Entry (yr)**	50.08 ± 5.87	49.88 ± 3.46	50.87 ± 5.38	47.29 ± 1.98	50.3 ± 4.7
	**Left entry**				
**Breast Area (cm** ^**2**^ **)**	121.53 ± 40.29	101.81 ± 26.08	88.43 ± 31.94	85.71 ± 40.56	100.28 ± 35.36
**- Dense Area (cm** ^**2**^ **)**	19.60± 10.45	35.57 ± 14.64	38.00 ± 19.10	35.77 ± 13.96	32.49 ± 16.42
**- Lucent Area (cm** ^**2**^ **)**	101.93 ± 39.07	66.24 ± 19.39	50.43 ± 19.91	49.95 ± 43.38	67.78 ± 35.01
**Overall PD (%)**	17.23 ± 10.70	34.91 ± 11.88	42.25 ± 15.65	48.15 ± 20.34	34.72 ± 17.54
	**Left exit**				
**Breast Area (cm** ^**2**^ **)**	125.38 ± 46.15	101.88 ± 25.53	89.29 ± 30.52	84.04 ± 45.60	101.25 ± 37.78
**- Dense Area (cm** ^**2**^ **)**	13.55 ± 7.92	33.35 ± 8.70	43.92 ± 16.83	54.63± 28.94	34.75 ± 20.34
**- Lucent Area (cm** ^**2**^ **)**	111.83 ± 45.03	68.54 ± 20.02	45.36 ± 15.11	29.41 ± 16.80	66.50 ± 38.93
**Overall PD (%)**	11.69 ± 5.97	33.14 ± 6.13	48.92 ± 5.58	65.46 ± 2.11	37.25 ± 18.71
	**Right exit**				
**Breast Area (cm** ^**2**^ **)**	119.08 ± 40.63	99.62 ± 26.70	86.76 ± 33.63	83.51 ± 45.96	98.18 ± 36.73
**- Dense Area (cm** ^**2**^ **)**	19.47 ± 18.51	29.73 ± 9.30	43.87 ± 17.31	33.78 ± 17.65	32.08± 17.61
**- Lucent Area (cm** ^**2**^ **)**	99.61 ± 45.39	69.88 ± 23.64	42.90 ± 17.55	49.73 ± 53.39	66.10 ± 39.41
**Overall PD (%)**	17.79 ± 14.07	30.83 ± 10.16	50.50 ± 6.39	50.29 ± 22.69	36.32 ± 18.28

**Fig 3:**
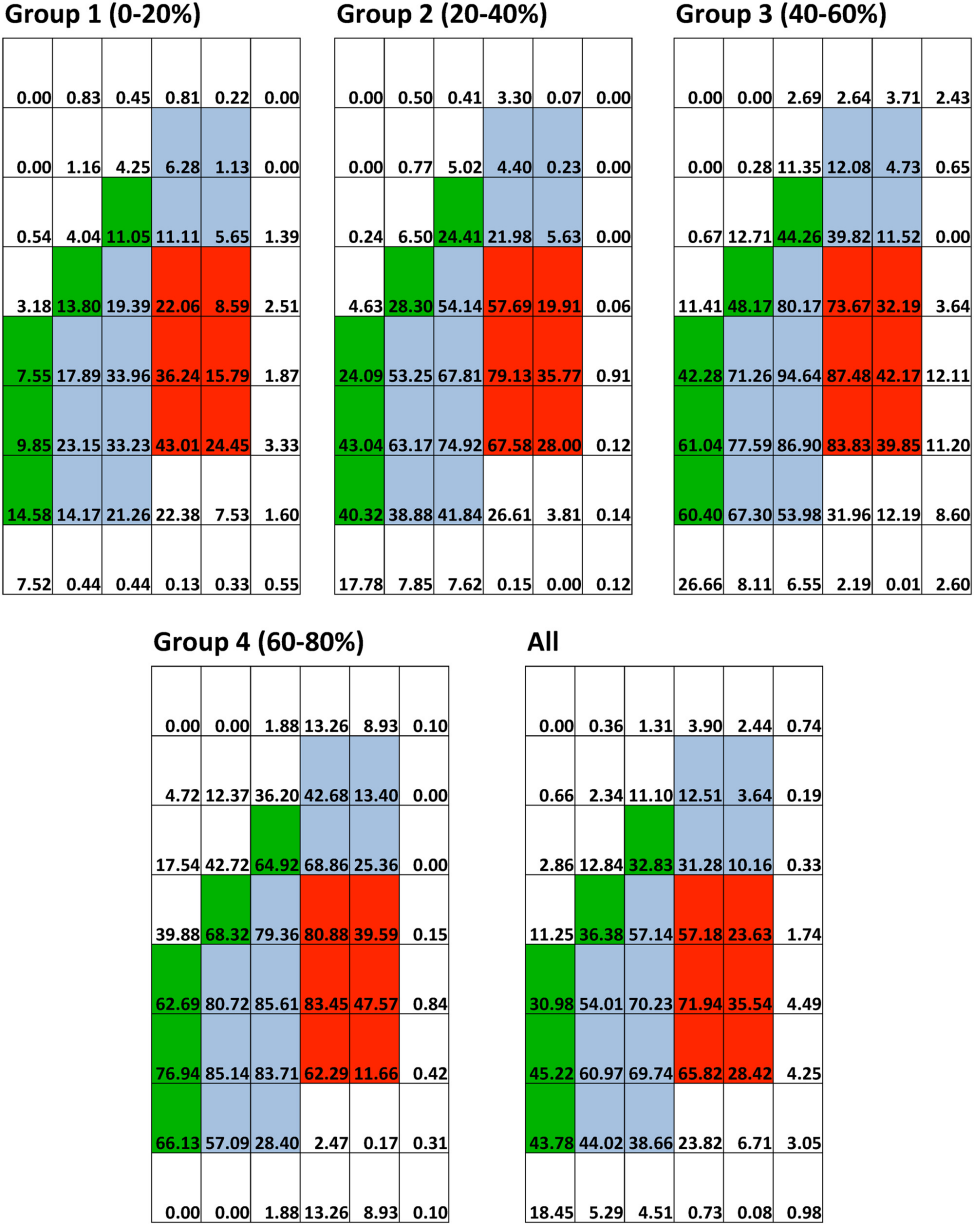
The spatial distribution pattern of regional PD in the present study. Numerical value inside the coordinate system represents the regional PD at each sub-region. Please note that the anterior, middle and posterior zones were highlighted in red, blue and green respectively.

The spatial distribution patterns of MBD in the 48 coordinates (regional PD) and in the 3 zones (zonal PD) were presented in Fig 3. Non-parametric Friedman test and post hoc Wilcoxon Signed-rank Test were used to test whether significant difference exists in zonal PD among the four groups. From Tables 2 and 3, our results indicated that in the less denser breasts groups (Group 1 and Group 2), MBD was clustered significantly and predominately at the anterior zone then followed by middle zone and posterior zone, but this spatial distribution pattern was not demonstrated in the denser breast groups (group 3 and 4).

**Table 2:** A summary of the zonal PDs of the left exit mammograms in the four groups.

	Zonal PD (%) at Posterior Zone	Zonal PD (%) at Middle Zone	Zonal PD (%) at Anterior Zone	Friedman test *(p*-value)
**Group 1 (<20%)**	11.36 ± 9.33	17.02 ± 12.14	25.02 ± 16.72	0.002
**Group 2(20–40%)**	32.03 ± 18.27	38.75 ± 12.23	48.01 ± 16.19	0.002
**Group 3(40–60%)**	51.23 ± 23.45	54.54 ± 10.56	59.86 ± 25.76	0.766
**Group 4(60–80%)**	67.80 ± 36.22	59.12 ± 27.26	54.24 ± 28.22	0.102
All	37.84 ± 28.38	41.12 ± 21.15	46.92 ± 24.63	0.007

**Table 3:** Post hoc comparisons of zonal PD in Group 1 and 2 using Wilcoxon Signed-rank Test.

	Post-hoc comparisons: Wilcoxon Signed-rank Test (*p*-value)		
	Posterior zone—Middle zone	Posterior zone—Anterior zone	Middle zone—Anterior zone
**Group 1 (<20%)**	0.003	0.023	0.050
**Group 2(20–40%)**	0.049	0.013	0.013
**All**	0.047	0.029	0.037

In this study, the Moran's I values were used to represent the spatial autocorrelation pattern of regional PD (Table 4). Moran's I value is ranged from -1 to +1, and is used to reflect the degree of spatial autocorrelation, and positive, zero and negative Moran's I values representing randomly clustered and dispersed patterns respectively. The Moran's I values in the present study were larger than zero, indicating that MSD in the breast of Chinese women have a positive spatial autocorrelations pattern. In general, there were no significant difference in Moran's I values between left and right breasts (*p* = 0.31), indicating that the spatial autocorrelations patterns of regional PD were similar in both sides of the breast. However, the Moran's I values at the left exit mammograms were significantly reduced at the left entry mammograms (*p* = 0.01), indicating the degree of positive spatial autocorrelations pattern could change with age in the Chinese population.

**Table 4:** A summary of Moran's I values in Left entry, Left exit and Right exit mammograms.

	Moran's I value				
	Group 1 (<20%)	Group 2 (20–40%)	Group 3 (40–60%)	Group 4 (60–80%)	All
**Left entry**	0.32 ± 0.07*	0.40 ± 0.07*	0.41 ± 0.10	0.38 ± 0.05*	0.38 ± 0.09*
**Left exit**	0.28 ± 0.09*	0.37 ± 0.10*	0.41 ± 0.06	0.36 ± 0.10*	0.36 ± 0.10*
**Right exit**	0.28 ± 0.11	0.37 ± 0.07	0.39 ± 0.07	0.37 ± 0.11	0.36 ± 0.10

* Denotes the Moran's I value at left entry mammograms were significantly reduced (p<0.01 using Wilcoxon signed-rank test) when compared to the left exit mammograms in different group.

We also found negative correlations between the sizes of breast (breast area) and overall PD at left entry, left exit and right exit mammograms (Spearman's rank correlation coefficient (*r)* = -0.36 to -0.52, all *p-* values < 0.01). This observation is consistent with a previous study [13]. Also, apart from a positive autocorrelation pattern in regional PD that was similar the Caucasian women, significant positive correlations between overall PD and Moran's I values were also found at left entry, left exit and right exit mammograms (Spearman's rank correlation coefficient (*r)* = 0.44 to 0.70, all *p-* values < 0.01), implying that the degree of MBD autocorrelation increased with its prevalence.

## Discussion and Conclusion

The present study defined spatial distribution and autocorrelation patterns of MBD with the application of a public domain Java-based image processing software, in an attempt to supplement the time-consuming and highly user dependent Culumus method [11,14,15] that is commonly used to dichotomize dense and non-dense breast tissues on mammograms for MBD detection. Nowadays, many computer algorithm approaches are highly correlated with the result generated by the gold standard method- Culumus method [11,14,16] in the segmentation of MBD, therefore the time and demands on experienced readers to perform segmentation of MBD are greatly reduced, resulting in an overall increase in productivity, reliability and reproducibility [11,13,14,17–20]. The only drawback of our proposed method is the requirement of an operator to outline the breast and remove the pectoral muscle. To solve this, the active contour model that developed by Ferrari et al. [21] can be integrated in our program for the contouring of breast and pectoral muscles automatically with improved accuracy [21,22]. Hence, the ultimate goal of a fully automated and high-throughput program is possible in the future. This study also has two limitations. As it is not mandatory for our referring doctors to document the history of hormonal therapy and diet preference of each patient, the effect of hormonal therapy and dietary factor on breast density fluctuation could not be determined.

From the finding of spatial distribution and autocorrelation patterns, the radio-dense tissues of the breast were generally not evenly distributed throughout the breast. For women with overall PD smaller than 40%, their radio-dense tissues were predominately clustered in the anterior zone, which was roughly the region just behind the nipple. This observation was consistent with the current biological knowledge in normal sized breasts where the majority of mammary glands is clustered behind nipples, and are the major radio-dense tissues.

In summary, the major findings of this study include (1) Chinese women with smaller breast size tend to have breast density clustered in the anterior part of the breast, but those with a larger breast size tend to have breast density clustered near the posterior part of the breast, (2) their left and right breasts were symmetric to each other in terms of the total breast area, overall PD and Moran's I values, and (3) a significant reduction in Moran's I value between the entry and exit mammograms was noted, indicating that their mammographic breast density tends to be less autocorrelated with age.

All in all, the knowledge gained from this study will be useful for further study on the distribution of radiodense cancer tissues in the breast and cancer risk. Despite the relative small sample size and lack of robust automated computation program in the segmentation and quantification of MBD, we established a semi-automated program for the segmentation and quantification of MBD, and gathered some pilot data about the spatial distribution and autocorrelation patterns of MBD that could provide additional insights into the effect of aging on MBD distribution in Chinese women. Further study is warranted using larger sample size and different racial groups.
